# Epidemiological profile of accidents involving venomous animals: a descriptive and time series study, Maranhão, 2014–2024

**DOI:** 10.1590/S2237-96222026v35e20251224.en

**Published:** 2026-08-03

**Authors:** Laryssa Stefany de Azevedo Santos, Helena de Paula Gonçalves Lima, Ismália Cassandra Costa Maia Dias, Marcelo Soares dos Santos, Marcelino Santos, Leonardo Hunaldo dos Santos

**Affiliations:** 1 Universidade Federal do Maranhão Centro de Ciências de Imperatriz Imperatriz MA Brazil Universidade Federal do Maranhão, Centro de Ciências de Imperatriz, Imperatriz, MA, Brazil; 2 Universidade Federal do Maranhão Centro de Ciências Sociais Imperatriz MA Brazil Universidade Federal do Maranhão, Centro de Ciências Sociais, da Saúde e Tecnologia, Imperatriz, MA, Brazil

**Keywords:** Environmental Health Surveillance, One Health, Environment and Public Health, Animals, Poisonous, Epidemiology, Vigilancia Sanitaria Ambiental, Salud Única, Medio Ambiente y Salud Pública, Animales Venenosos, Epidemiología

## Abstract

**Objective:**

To analyze the epidemiological profile and temporal trend of accidents involving venomous animals in Maranhão, from 2014 to 2024.

**Methods:**

A descriptive and ecological study with time-series analysis aggregated by Health Regions, using data on accidents involving venomous animals obtained from the Notifiable Diseases Information System (*Sistema de Informação de Agravos de Notificação*, SINAN). The epidemiological profile was analyzed using absolute and relative frequencies, with incidence rates calculated by venomous animal group. Temporal trend analysis was conducted using the Prais-Winsten linear regression model to estimate the annual percent change (APC) with its 95% confidence interval (95%CI).

**Results:**

A total of 44,620 cases of accidents involving venomous animals were recorded in Maranhão. Ophidism was the most frequent (46.4%), with higher occurrence among males (76.3%), individuals aged 20 to 39 years (34.2%), those self-identified as Brown (Brazilian mixed race) (73.0%), and those with incomplete elementary education (32.4%). In the state, the temporal trend was stable for ophidism (APC 2.50; 95%CI -0.10; 5.16) and erucism (APC -0.71; 95%CI -3.67; 2.35), whereas scorpionism (APC 7.94; 95%CI 4.75; 11.23), arachnidism (APC 7.87; 95%CI 6.37; 9.40), and bee stings (APC 4.47; 95%CI 1.13; 7.92) showed increasing trends.

**Conclusion:**

Accidents involving venomous animals predominantly affected men, young individuals, those self-identified as Brown, and individuals with low educational levels. The temporal trend indicated a growing concern, particularly regarding scorpionism. Overall, the findings reinforce the need for continuous surveillance and regionally tailored public policies for prevention, integrating epidemiological, environmental, and health education actions.

## Resumen

**Objetivo:**

Analizar el perfil epidemiológico y la tendencia temporal de los accidentes causados por animales venenosos en Maranhão durante el período 2014–2024.

**Métodos:**

Estudio descriptivo y ecológico con análisis de series temporales agregadas por Regiones de Salud, utilizando datos sobre accidentes por animales venenosos obtenidos del Sistema de Información de Enfermedades de Notificación Obligatoria (*Sistema de Informação de Agravos de Notificação*, SINAN). El perfil epidemiológico se analizó mediante frecuencias absolutas y relativas y se calcularon tasas de incidencia según el tipo de animal venenoso. La tendencia temporal se evaluó mediante el modelo de regresión lineal de Prais-Winsten, estimando la variación porcentual anual (VPA) con sus respectivos intervalos de confianza del 95% (IC95%).

**Resultados:**

Se registraron 44.620 casos de accidentes causados por animales venenosos en Maranhão. El ofidismo fue el más frecuente (46,4%), con mayor ocurrencia en hombres (76,3%), personas de 20 a 39 años (34,2%), individuos pardos (73,0%) y con educación primaria incompleta (32,4%). A nivel estatal, la tendencia temporal fue estable para el ofidismo (VPA 2,50; IC95% -0,10; 5,16) y para el erucismo (VPA -0,71; IC95% -3,67; 2,35), mientras que el escorpionismo (VPA 7,94; IC95% 4,75; 11,23), el araneismo (VPA 7,87; IC95% 6,37; 9,40) y los accidentes por picadura de abejas (VPA 4,47; IC95% 1,13; 7,92) mostraron una tendencia creciente.

**Conclusión:**

Los accidentes por animales venenosos afectaron predominantemente a hombres, a personas jóvenes, a personas pardas y a personas con baja escolaridad. Las tendencias temporales indican un escenario de creciente preocupación, especialmente en lo que respecta al escorpionismo. En general, los hallazgos refuerzan la necesidad de vigilancia continua y de políticas públicas de prevención regionalizadas, que integren acciones epidemiológicas, ambientales y de educación en salud.

**Ethical aspects**

This research used public domain anonymized databases.

## Introduction

Accidents caused by venomous animals constitute an important public health problem in Brazil, with direct implications for morbidity and mortality, especially among socioeconomically vulnerable populations. Their occurrence is associated with several risk factors, such as the encroachment of human activities into natural areas and the unplanned expansion of urban and agricultural areas, compounded by the lack of effective public policies for prevention and control [1,2].

In the context of global health, these accidents were included in the World Health Organization (WHO) list of Neglected Tropical Diseases (NTD) in 2007. Although they were removed from this classification in 2013, ophidism was reintroduced in 2017, given its high morbidity and mortality burden and the vulnerability of affected populations [2,3].

In Brazil, accidents caused by venomous animals are notifiable health conditions, according to the guidelines of the National Health Surveillance System (*Sistema Nacional de Vigilância em Saúde*, SNVS) and the National Health Surveillance Policy (*Política Nacional de Vigilância em Saúde*, PNVS) [1,4]. Surveillance of these events is strategic for reducing morbidity and mortality. In 2015, the country established the goal of reducing deaths associated with these conditions by 50% through strengthening health care, ensuring adequate antivenom distribution, and organizing a coordinated health services response [1].

Between 2007 and 2021, approximately 2,693,706 accidents caused by venomous animals were recorded in Brazil, with an incidence of 88.9 cases per 100,000 inhabitants. The Northeast region accounted for a substantial portion of this burden, with an incidence of 105.5 cases per 100,000 inhabitants. In Maranhão, during the same period, 44,306 cases were reported, with a mean incidence of 41.13 cases per 100,000 inhabitants. These data highlight the relevance of this condition in the state context [5].

Maranhão presents ecological and territorial characteristics that favor the occurrence of these accidents. The state encompasses transition areas between the Amazon, Cerrado, and Caatinga biomes, characterized by strong climatic seasonality and diverse land-use patterns [6,7]. In this context, it is observed the presence of several genera of venomous animals of medical importance, such as the jararacas (Bothrops), rattlesnakes (Crotalus), surucucus (Lachesis), coral snakes (Micrurus), scorpions (Tityus), Brazilian wandering spiders (Phoneutria), black or brown widow spiders (Latrodectus) and brown spiders (Loxosceles) [1]. 

Many of these species exhibit synanthropic behavior, that is, they adapt to urban and peri-urban environments that provide shelter and food, favoring their proliferation [1,8]. This risk is further accelerated by urbanization. Between 1960 and 2010, the urban population of Maranhão grew by approximately 924%, accompanied by the expansion in the number of municipalities [9]. Associated with deforestation and habitat fragmentation, these processes intensify human–animal contact and increase the risk of accidents [1].

Given the magnitude and territorial heterogeneity of this condition, approaches based on Health Regions are essential for identifying intrastate inequalities and supporting decentralized actions in surveillance, prevention, and health care [10]. In this sense, time series analysis is an essential tool for identifying trends and seasonal patterns, especially in conditions influenced by ecological and climatic determinants [11].

Therefore, the present study aimed to analyze the epidemiological profile and temporal trends of accidents involving venomous animals in the state of Maranhão from 2014 to 2024.

## Methods

### Study design 

This is a descriptive and ecological study with time series analysis aggregated by Health Regions of Maranhão, covering the period from 2014 to 2024.

### Data sources and information processing 

The microdata were extracted in October 2025 through the Tabwin system, from the files of the Notifiable Diseases Information System (*Sistema de Informação de Agravos de Notificação*, SINAN). The files, initially in .dbc format, were converted and processed in the RStudio software, using the variable dictionary and the reporting form for accidents involving venomous animals to decode the observations. The data used are available in the SciELO Data repository [13].

Duplicate record analyses were not performed because the data made available by SINAN are anonymized and aggregated and do not include a specific categorization that allows verification of duplicate records. Population estimates were obtained from the Brazilian Institute of Geography and Statistics (*Instituto Brasileiro de Geografia e Estatística*, IBGE) through the IBGE Automatic Recovery System (*Sistema IBGE de Recuperação Automática*, SIDRA). The resident population of Maranhão was used for statistical purposes [12], with population data matched to the respective year of occurrence of the accidents, ensuring temporal consistency in the calculation of rates.

### Setting

Maranhão is located in the Northeast macroregion of Brazil, covering a territorial area of 329,651.5 km^2^ and an estimated population of 6,776,699 inhabitants. Administratively, the state is divided into 217 municipalities [12] and structured into 19 Health Regions. These play a strategic role in the management of the Unified Health System (Brazilian Unified Health System, SUS), organizing, planning, and operating health actions and services at the regional level (Figure 1) [10].

**Figure 1:**
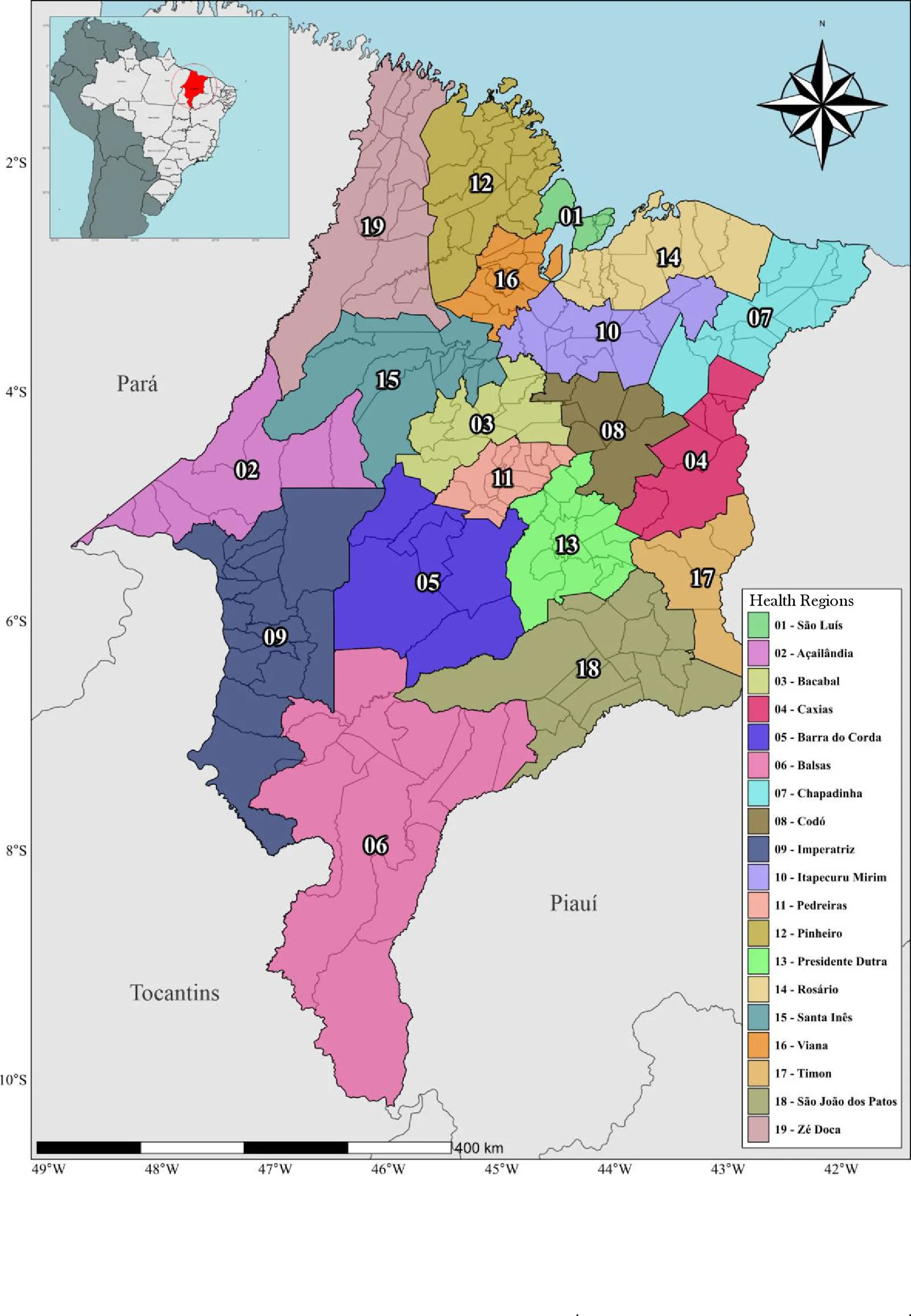
Territorial division of Maranhão, showing the distribution of municipalities and Health Regions

The territorial organization of the Health Regions considered in this study followed the current administrative organization of Maranhão, consolidated according to the guidelines of Tripartite Intergovernmental Commission Resolution (*Resolução Comissão Intergestores Tripartite*, CIT) No. 37/2018 and formalized by Bipartite Intergovernmental Commission Resolution (*Resolução Comissão Intergestores Bipartite*, CIB) No. 64/2018, which organizes the state into 19 Health Regions [10]. Considering that SINAN records are produced at the municipal level, aggregation by Health Regions was adopted exclusively for analytical purposes and does not compromise the comparability of the historical series, even though administrative reorganizations occurred during the study period.

### Population

All records of accidents involving venomous animals reported to SINAN, occurring between 2014 and 2024, whose municipality of occurrence was located in Maranhão, were included. These records were used exclusively to identify and subsequently aggregate them into their respective Health Regions, which constituted the unit of analysis for the study. Records classified as “other venomous animals,” including accidents caused by amphibians, centipedes, dogs, goats, cats, horses, and others, were excluded. This exclusion was adopted due to the heterogeneity of the category and the limitation for specific analyses by animal group.

### Period

The inclusion of the year 2014 allowed the construction of a baseline before the implementation of the Sustainable Development Goals, initiated in 2015, enabling the evaluation of trends over an extended period. It was possible to assess trends over an extended period. The interval up to 2024 was maintained to encompass the most recent available historical series. However, it is acknowledged that the years 2020 and 2021, which correspond to the COVID-19 pandemic period, may have influenced reporting patterns.

### Variables

The variables considered essential for the epidemiological analysis were the causative animal agent, year, municipality of occurrence, sex, age group, race/skin color, and educational level. These variables presented high completion rates throughout the historical series. Data quality was evaluated based on variable completeness and the maintenance of conceptual definitions and data entry structure over the analyzed period.

The stratifications included in the analysis were:

a) Year of accident occurrence (2014 to 2024);
b) State of occurrence: Maranhão;
c) Health Regions (Açailândia; Bacabal; Balsas; Barra do Corda; Caxias; Chapadinha; Codó; Imperatriz; Itapecuru Mirim; Pedreiras; Pinheiro; Presidente Dutra; Rosário; Santa Inês; São João dos Patos; São Luís; Timon; Viana; Zé Doca);
d) Gender (male, female, ignored/missing);
e) Age range(<1, 1–4, 5–9, 10–14, 15–19, 20–39, 40–59, 60–64, 65–69, 70–79, ≥80, ignored/missing);
f) Race/skin color (White; Black; Brown [Brazilian mixed race]; Asian; Indigenous)
g) Educational level (not applicable, illiterate, incomplete elementary education, complete elementary education, incomplete secondary education, complete secondary education, incomplete higher education, complete higher education, ignored/missing);
h) Type of accident (snake, scorpion, spider, bee, caterpillar).

### Statistical methods

Descriptive analysis was conducted by calculating absolute and relative frequencies of the selected variables. Annual incidence rates per 100,000 inhabitants were calculated for each type of accident and Health Region, according to the formula:


1 $$\mathrm{IR}=\left(\frac{C_{\mathrm{HR}}}{P_{\mathrm{HR}}}\right)\times 100,000$$

Where: 

IR – incidence rate per 100,000 inhabitants;

$C_{\mathrm{HR}}$ – Number of accidents involving venomous animals by Health Region and year;

$P_{\mathrm{HR}}$ – Resident population exposed by Health Region and year.

Temporal trend analysis of the rates was conducted using the Prais-Winsten linear regression model, with first-order serial autocorrelation (AR(1)) correction, applied to incidence rates previously transformed into decimal logarithms, for each type of accident, considering the 19 Health Regions as ecological units. 

From the coefficients obtained in the regression, the annual percent change (APC) and its respective 95% confidence intervals (95%CI) were estimated, according to the formulas below [8]:


$$\mathrm{APC}=\left[-1+10^{b1}\right]\times 100\%$$

$${95\mathrm{\%CI}}_{\mathrm{lower}}=\left[-1+\;10^{b1,\min}\right]\times 100\%$$

$${95\mathrm{\%CI}}_{\mathrm{upper}}=\left[-1+\;10^{b1,\max}\right]\times 100\%$$

Trends were interpreted according to the following criteria [8]:

- Increasing: positive change, with 95%CI not including the null value;
- Decreasing: negative change, with 95%CI not including the null value;
- Stable: change with a 95%CI that includes the null value.

All analyses were conducted using RStudio software, version 4.5.0, with the prais-package function [14].

## Results

In Maranhão, between 2014 and 2024, a total of 44,620 cases of accidents involving venomous animals were reported. Ophidism was the most frequent form (46.4%), followed by scorpionism (40.7%), Arachnidism (6.0%), bee stings (5.0%), and erucism (1.9%). Overall, accidents involving venomous animals mainly affected men (65.0%), individuals aged 20 to 39 years (34.5%), those self-identified as Brown (76.7%), and individuals with low educational level (28.8%).

Ophidism presented the highest incidence (33.4/100,000 inhabitants) during the analyzed period (Figure 2). The predominant profile consisted of men (76.3%), adults aged 20 to 39 years (34.2%), Brown individuals (73.0%), and those with low educational level (32.4%) (Table 1). Regarding the temporal trend, increases were observed in some Health Regions, particularly Balsas (APC 4.04; 95%CI 2.04; 6.07), Pinheiro (APC 7.65; 95%CI 4.93; 10.43), Rosário (APC 5.38; 95%CI 3.40; 7.40), and Viana (APC 4.14; 95%CI 1.68; 6.66). However, for the state as a whole, the trend was stable (APC 2.50; 95%CI -0.10; 5.16) (Table 2, Figure 2).

**Table 2:** Annual percent change (APC) and 95% confidence interval (95%CI) of the incidence rate (per 100,000 inhabitants) by type of accident and Health Regions. Maranhão, 2014–2024 (n=44,620)

Health Regions	Ophidism APC (95%CI)	Scorpionism APC (95%CI)	Arachnidism APC (95%CI)	Bee sting APC (95%CI)	Erucism APC (95%CI)
Açailândia	0.70 (-0.24; 1.64)	10.23 (7.38; 13.17)	7.24 (4.94; 9.60)	8.85 (-0.22; 18.74)	2.05 (-6.93; 11.89)
Bacabal	0.98 (-2.38; 4.45)	12.25 (5.89; 18.98)	10.80 (8.57; 13.09)	5.23 (-2.64; 13.73)	1.96 (-5.22; 9.69)
Balsas	4.04 (2.04; 6.07)	11.90 (5.06; 19.18)	8.63 (3.02; 14.54)	4.11 (-1.00; 9.49)	-5.57 (-12.44; 1.84)
Barra do Corda	3.85 (-1.71; 9.72)	6.83 (1.23; 12.74)	6.58 (0.31; 13.24)	11.84 (2.96; 21.48)	-0.28 (-7.11; 7.06)
Caxias	0.81 (-4.20; 6.09)	4.03 (1.29; 6.83)	5.02 (-0.46; 10.81)	9.71 (4.17; 15.54)	4.42 (0.98; 7.97)
Chapadinha	2.29 (-0.26; 4.89)	3.37 (0.27; 6.57)	14.45 (8.56; 20.67)	-1.28 (-3.57; 1.07)	11.26 (7.75; 14.88)
Codó	1.07 (-5.28; 7.84)	6.41 (3.24; 9.68)	8.93 (6.69; 11.22)	15.51 (10.02; 21.28)	1.62 (-4.94; 8.62)
Imperatriz	0.42 (-1.68; 2.55)	7.55 (5.67; 9.47)	11.03 (6.56; 15.70)	11.27 (6.00; 16.80)	-3.34 (-7.55; 1.06)
Itapecuru Mirim	1.70 (-0.19; 3.63)	1.67 (-0.46; 3.84)	4.76 (-0.52; 10.32)	10.75 (4.43; 17.44)	7.70 (-2.42; 18.87)
Pedreiras	-0.53 (-3.23; 2.24)	12.68 (7.03; 18.63)	6.23 (0.12; 12.71)	0.03 (-7.28; 7.92)	-2.90 (-5.37; -0.36)
Pinheiro	7.65 (4.93; 10.43)	9.38 (1.10; 18.34)	11.24 (6.48; 16.21)	2.54 (-1.64; 6.90)	-2.91 (-8.88; 3.45)
Presidente Dutra	1.00 (-3.72; 5.95)	3.76 (1.14; 6.44)	7.68 (5.86; 9.53)	6.51 (-1.74; 15.45)	-0.88 (-12.25; 11.96)
Rosário	5.38 (3.40; 7.40)	3.81 (2.19; 5.45)	4.08 (1.20; 7.05)	21.99 (14.85; 29.57)	1.53 (1.53; 1.53)
Santa Inês	1.90 (-1.49; 5.41)	12.47 (8.36; 16.74)	12.18 (6.15; 18.54)	9.26 (-6.14; 27.18)	-8.96 (-23.80; 8.78)
São João dos Patos	-0.82 (-4.04; 2.50)	6.56 (2.77; 10.49)	9.78 (3.57; 16.37)	-14.79 (-18.15; -11.30)	5.15 (-0.06; 10.63)
São Luís	3.64 (-0.21; 7.63)	6.76 (2.33; 11.39)	4.87 (2.74; 7.04)	0.15 (-5.66; 6.33)	2.83 (-4.96; 11.25)
Timon	3.73 (-1.64; 9.39)	11.68 (2.63; 21.52)	7.55 (3.33; 11.95)	-9.24 (-20.86; 4.08)	1.29 (1.29; 1.29)
Viana	4.14 (1.68; 6.66)	12.45 (6.23; 19.04)	5.14 (3.90; 6.39)	0.37 (0.37; 0.37)	4.94 (-14.39; 28.63)
Zé Doca	0.63 (-2.12; 3.46)	10.35 (3.51; 17.64)	3.04 (-2.28; 8.64)	-6.29 (-8.19; -4.35)	-24.58 (-24.58; -24.58)
Maranhão	2.50 (-0.10; 5.16)	7.94 (4.75; 11.23)	7.87 (6.37; 9.40)	4.47 (1.13; 7.92)	-0.71 (-3.67; 2.35)

**Table 1:** Absolute frequency (n) and percentage (%) of accidents caused by venomous animals, according to sociodemographic variables. Maranhão, 2014–2024 (n=44,620)

Variable	Ophidism n (%)	Scorpionism n (%)	Arachnidism n (%)	Bee sting n (%)	Erucism n (%)
Sex					
Male	15,822 (76.3)	9,875 (54.4)	1,348 (50.1)	1,462 (65.9)	486 (58.0)
Female	4,904 (23.7)	8,272 (45.6)	1,343 (49.9)	756 (34.1)	352 (42.0)
**Age group ** (years)					
<1	370 (1.8)	464 (2.6)	89 (3.3)	92 (4.1)	49 (5.8)
1–4	363 (1.8)	533 (2.9)	61 (2.3)	167 (7.5)	81 (9.7)
5–9	1,158 (5.6)	881 (4.9)	95 (3.5)	209 (9.4)	119 (14.2)
10–14	1,725 (8.3)	1,087 (6.0)	117 (4.3)	153 (6.9)	101 (12.1)
15–19	2,186 (10.5)	1,463 (8.1)	175 (6.5)	180 (8.1)	45 (5.4)
20–39	7,098 (34.2)	6,241 (34.4)	1,093 (40.6)	729 (32.9)	215 (25.7)
40–59	5,458 (26.3)	4,997 (27.5)	744 (27.6)	486 (21.9)	155 (18.5)
60–64	879 (4.2)	891 (4.9)	131 (4.9)	70 (3.2)	24 (2.9)
65–69	632 (3.0)	684 (3.8)	72 (2.7)	44 (2.0)	16 (1.9)
70–79	685 (3.3)	695 (3.8)	80 (3.0)	51 (2.3)	29 (3.5)
≥80	172 (1.0)	211 (1.1)	34 (1.3)	37 (1.7)	4 (0.5)
**Race/skin color**					
Brown (Brazilian mixed race)	15,124 (73.0)	14,503 (79.9)	2,101 (78.1)	1,833 (82.6)	679 (81.0)
Indigenous	1,834 (8.8)	132 (0.7)	22 (0.8)	13 (0.6)	4 (0.5)
Black	1,716 (8.3)	1,328 (7.3)	166 (6.2)	101 (4.6)	61 (7.3)
White	1,336 (6.4)	1,475 (8.1)	287 (10.7)	183 (8.3)	67 (8.0)
Asian	199 (1.0)	215 (1.2)	12 (0.4)	10 (0.5)	5 (0.6)
Ignored/blank	517 (2.5)	494 (2.7)	103 (3.8)	78 (3.5)	22 (2.6)
**Educational level**					
Not applicable	1,117 (5.4)	1,331 (7.3)	188 (7.0)	355 (16.0)	167 (19.9)
Illiterate	1,575 (7.6)	1,013 (5.6)	91 (3.4)	78 (3.5)	25 (3.0)
Incomplete elementary education	6,716 (32.4)	4,790 (26.4)	522 (19.4)	568 (25.6)	252 (30.1)
Complete elementary education	2,637 (12.7)	2,019 (11.1)	249 (9.2)	222 (10.0)	74 (8.8)
Incomplete high school	1,137 (5.5)	1,207 (6.7)	177 (6.6)	111 (5.0)	33 (3.9)
Complete high school	1,445 (7.0)	2,591 (14.3)	475 (17.7)	347 (15.6)	91 (10.9)
Incomplete higher education	69 (0.3)	128 (0.7)	50 (1.8)	20 (1.0)	9 (1.1)
Complete higher education	137 (0.7)	313 (1.7)	104 (3.9)	52 (2.3)	16 (1.9)
Ignored/blank	5,893 (28.4)	4,755 (26.2)	835 (31.0)	465 (21.0)	171 (20.4)

**Figure 2:**
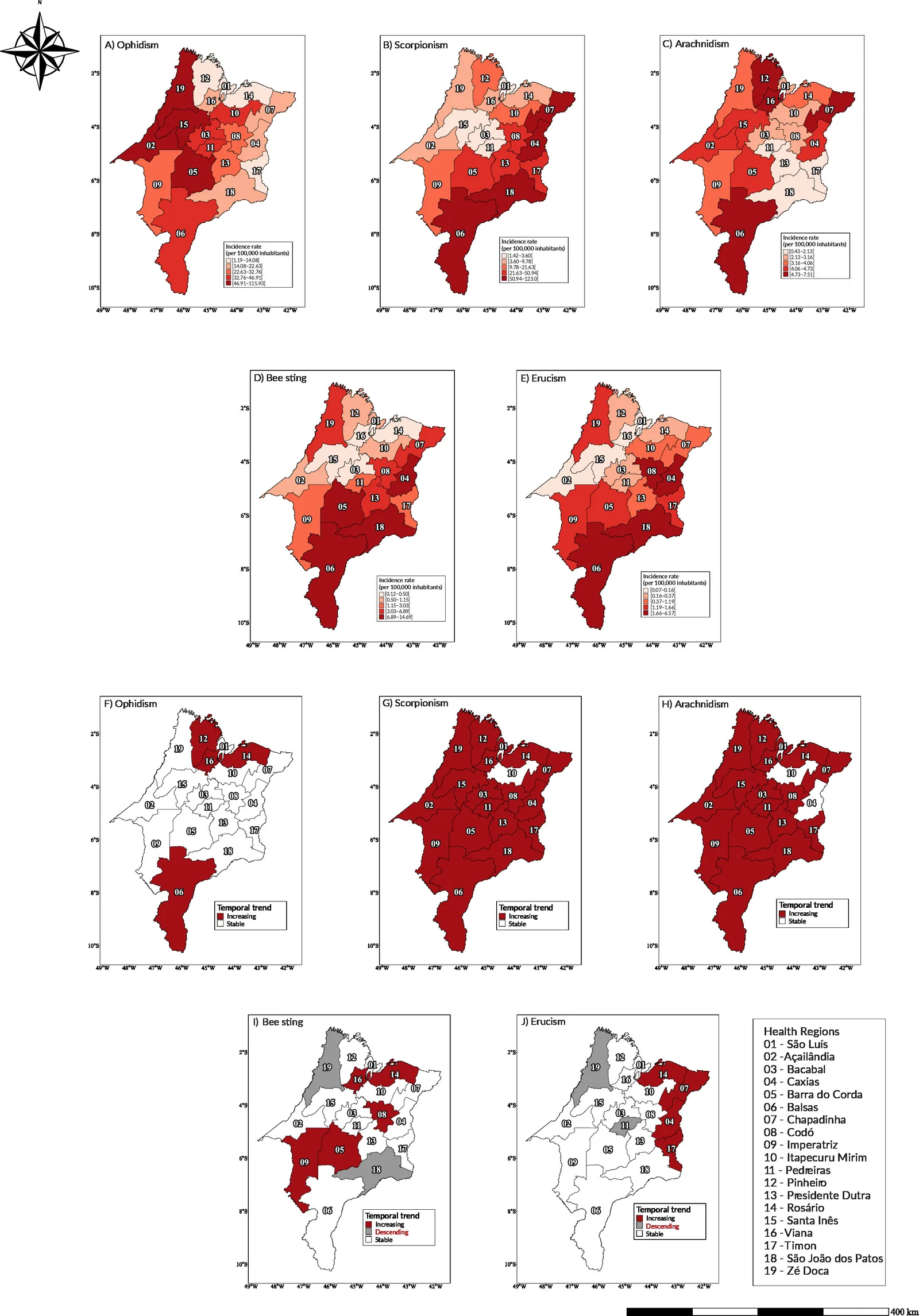
Incidence of accidents caused by venomous animals by type of condition: (A) ophidism; (B) scorpionism; (C) arachnidism; (D) bee sting; (E) erucism; (F) ophidism; (G) scorpionism; (H) arachnidism; (I) bee sting; (J) erucism, by Health Regions. Maranhão, 2014–2024

Scorpionism was the second most frequent condition, with an incidence (29.6/100,000 inhabitants) close to that observed for ophidism (Figure 2). The sociodemographic profile was similar to that observed for ophidism, with a slight predominance of males (54.4%), occurrence among adults aged 20 to 39 years (34.4%), Brown individuals (79.9%), and individuals with low educational level (26.4%) (Table 1). Temporal analysis indicated an increasing trend in Maranhão (APC 7.94; 95%CI 4.75; 11.23), with growth observed in most Health Regions and stability only in Itapecuru Mirim (APC 1.67; 95%CI -0.46; 3.38) (Table 2, Figure 2).

Arachnidism presented lower incidence (3.5/100,000 inhabitants) (Figure 2) and frequency, with a relatively balanced distribution between males (50.1%) and females (49.9%), and higher occurrence among individuals aged 20 to 39 years (40.6%), Brown individuals (78.1%), and those with low educational level (19.4%) (Table 2). However, a growing temporal trend was observed in most Health Regions, resulting in an overall increase for the state (APC 7.87; 95%CI 6.37; 9.40) and stability in Caxias (APC 5.05; 95%CI −0.46; 10.81), Itapecuru Mirim (APC 4.76; 95%CI −0.52; 10.32), and Zé Doca (APC 3.04; 95%CI −2.28; 8.64) (Table 2, Figure 2). 

Bee stings presented a low incidence (3.7/100,000 inhabitants) (Figure 2) and predominance among males (65.9%), mainly affecting adults aged 20 to 39 years (32.9%), Brown individuals (82.6%), and those with low educational level (25.6%) (Table 1). The trend was increasing for Maranhão (APC 4.47; 95%CI 1.13; 7.92), with increases in several Health Regions, particularly Barra do Corda (APC 11.84; 95%CI 2.96; 21.48), Caxias (APC 9.71; 95%CI 4.17; 15.54), Codó (APC 15.51; 95%CI 10.02; 21.28), Imperatriz (APC 11.27; 95%CI 6.00; 16.80), Itapecuru Mirim (APC 10.75; 95%CI 4.43; 17.44), Rosário (APC 21.99; 95%CI 14.85; 29.57), and Viana (APC 0.37; 95%CI 0.37; 0.37), and a decreasing trend in São João dos Patos (APC −14.79; 95%CI −18.15; −11.30) and Zé Doca (APC −6.29; 95%CI −8.19; −4.35) (Table 2, Figure 2).

Erucism was the least frequent condition during the analyzed period, with low incidence (1.4/100,000 inhabitants) (Figure 2) and a slight predominance among males (58.0%), affecting individuals aged 20 to 39 years (25.7%), Brown individuals (81.0%), and those with low educational attainment (30.1%). It presented a more restricted distribution compared with the other types of accidents (Table 1), with increasing trends in Caxias (APC 4.42; 95%CI 0.98; 7.97), Chapadinha (APC 11.26; 95%CI 7.75; 14.88), Rosário (APC 1.53; 95%CI 1.53; 1.53), and Timon (APC 1.29; 95%CI 1.29; 1.29), and decreasing trends in Pedreiras (APC −2.90; 95%CI −5.37; −0.36) and Zé Doca (APC −24.58; 95%CI −24.58; −24.58), with stability for Maranhão (APC −0.71; 95%CI −3.67; 2.35) (Table 2, Figure 2).

## Discussion

Accidents caused by venomous animals in Maranhão exhibited an epidemiological pattern characterized by the coexistence of multiple conditions. A predominance of ophidism (46%) was observed, with a substantial incidence of scorpionism (41%). This profile reflects the state’s ecological, climatic, and territorial diversity, as well as the heterogeneity of human settlement processes. The unequal distribution of these conditions across the Health Regions highlights the influence of environmental, socioeconomic, and land-use factors on the dynamics of these accidents, highlighting the need for region-specific analyses and geographically targeted interventions.

From a sociodemographic perspective, a higher frequency of victims was observed among males and individuals of working age. This pattern is widely described in the national and international literature [15,16], especially in the case of ophidism. A direct relationship with rural work is noteworthy, with rural work predominantly performed by men (76%) who mainly reside in rural areas (76%), since agricultural activities and manual soil handling increase the risk of exposure to snakes[1,17].

The higher proportion of victims self-identified as Brown (76%) and with low educational level (28%) cannot be explained solely by the demographic composition of the state, indicating the influence of historically structured social and institutional inequalities. The differential vulnerability resulting from the accumulation and interaction of socioeconomic determinants modulates the risk of illness and its outcomes. Populations in vulnerable situations tend to reside in areas with precarious housing conditions, inadequate basic sanitation, and limited access to health services [18]. These conditions favor the presence of venomous animals in household and peridomestic environments and increase the risk of human contact, particularly in contexts where access to information and preventive measures is limited.

Although each condition presents its own dynamics, all are influenced by common socioenvironmental determinants. Ophidism occurs predominantly in rural areas (76%) and is often associated with field work [1,17]. The risk is increased in contexts involving contact with residues of bean, maize, or sugarcane crops, particularly affecting farmers, rubber tappers, fishers, Brazil-nut collectors, traditional communities, and Indigenous peoples, who often live far from urban centers and health services. In addition, traditional treatment practices, such as the use of tourniquets and the application of homemade substances (coffee grounds, vinegar, or urine), are common in relation to these accidents [1], which hinders the search for appropriate care and contributes to the underreporting of cases.

In Maranhão, the main snakes of importance in health belong to the genera jararaca (Bothrops), rattlesnake (Crotalus), surucucu (Lachesis) and coral (Micrurus), including common lancehead (*Bothrops atrox*), jararaca or surucucurana (Brazilian lancehead) (*Bothrops moojeni*), jararaca-do-rabo-branco (*Bothrops lutzi*), South American rattlesnake or boicininga (*Crotalus durissus cascavella*), surucucu-pico-de-jaca (South American bushmaster) (*Lachesis muta*), Brazilian short-tailed coral snake (*Micrurus brasiliensis*), thread coral snake or boicorá (*Micrurus filiformis*) and South American coral snake (*Micrurus lemniscatus*), among others [1]. Despite this diversity, the epidemiological relevance of these species is not homogeneous, as ecological and behavioral differences influence the risk of contact with the human population. In this context, the common lancehead (B. atrox) stands out for its high capacity to adapt to anthropized areas. It is frequently attracted by the presence of rodents, which favors its occurrence in domestic and peridomestic environments, and it is responsible for 70% of ophidism cases in Brazil [1].

Although ophidism accounted for the highest number of cases in Maranhão (46%), this pattern is not reflected at the national level. In Brazil, in 2022 (183,738 cases) and 2023 (202,714 cases), scorpionism was the main form of accidents involving venomous animals [1,16,19]. In 2023, scorpionism (202,714 cases) largely exceeded ophidism (32,595 cases) [17,19], especially in densely populated urban areas that offer favorable conditions for their proliferation [1]. In contrast, the greater incidence of ophidism in the context of Maranhão suggests the existence of local eco-epidemiological specificities associated with the extensive rural area of the state and with the intensification of the agricultural frontier in southern Maranhão, a region included in MATOPIBA (a region encompassing areas of Maranhão, Tocantins, Piauí, and Bahia) [20].

The increasing temporal trend in scorpionism (APC 7.94; 95%CI 4.75; 11.23), observed across most Health Regions, follows the pattern described in the national literature. This behavior has been strongly associated with accelerated and disorderly urbanization [21]. In Brazil, scorpion accidents are mostly caused by different species of the genus Tityus [1,16]. In Maranhão, with recorded distribution, the species *Tityus obscurus* and *Jaguajir agamenon* stand out, both widely distributed in the Northeast and North regions of the country [1]. In general, species of the genus Tityus exhibit synanthropic behavior, high ecological plasticity, and great capacity to adapt to urban environments. These characteristics favor their proliferation in places with accumulation of household waste, old buildings, vacant lots, and poor basic sanitation conditions [22]. Such conditions are frequently described in urban centers and peri-urban areas of Maranhão [23].

In Maranhão, three genera of spiders of public health importance are present: brown spiders (Loxosceles), black or brown widow spiders (Latrodectus), and Brazilian armed spiders (Phoneutria). Species of the genera Loxosceles and Latrodectus show good adaptation to domestic and peridomestic environments, where they are frequently found in debris, clothing, and footwear. Accidents generally occur when the animal is compressed against the body. Spiders of the genus Phoneutria, in contrast, exhibit more aggressive behavior and lower adaptation to urban environments. They are associated with areas with greater vegetation cover, especially palm and bromeliad leaves. In these cases, accidents occur while spiders move for foraging and reproduction, or when victims handle objects in the natural environment [1].

Bee stings showed an increasing trend (APC 4.47; 95%CI 1.13; 7.92), while erucism showed temporal stability (APC −0.71; 95%CI −3.67; 2.35), corresponding to the lowest incidences observed. Africanized bees (*Apis mellifera*) are widely distributed in urban and rural areas, and the severity of stings is generally associated with multiple stings. In 2023, bee stings accounted for 8% of the total accidents caused by venomous animals. As most accidents involve only a single bee, the total number of such events may be underreported [24].

From 2019 to 2023, erucism, mainly caused by caterpillars of the genus Lonomia, showed higher incidence in the South (5.90/100,000 inhabitants) and Southeast (4.43/100,000 inhabitants) regions of Brazil [25], a pattern associated with specific climatic and environmental conditions. However, processes such as climate change, deforestation, and increased urban lighting have favored the geographic expansion of these species into newly urbanized areas [1,25]. These caterpillars occur in both urban and rural areas and are currently considered synanthropic. Accidents commonly occur during human contact with tree trunks close to the ground. The resemblance of caterpillars to tree bark makes visual identification difficult, thereby favoring accidental contact and accidents [1].

Seasonality and rainfall patterns play a significant role in the occurrence of accidents involving venomous animals. In general, higher frequency is observed between October and April. The increase in ophidism and scorpion stings occurs mainly during the rainy season, when these animals exhibit greater biological activity [1]. In addition, human displacement in flooded areas becomes more frequent during these periods, increasing the risk of exposure. 

Projections related to climate change and environmental suitability suggest changes in the spatial distribution and behavior of venomous animals, particularly scorpions and spiders [26]. Climate modeling indicates potential effects of environmental changes on venomous snakes, including the redistribution of climatically suitable areas. Climate projections indicate that, by 2070, several species of venomous snakes may lose potentially suitable areas for their survival, while other regions, such as Africa and Asia, may become more vulnerable to the occurrence of these animals, with possible repercussions for accident risk patterns [27]. These processes contribute to recent ecological changes driven by deforestation and urban expansion, which favor ecological adaptations, particularly among synanthropic species [1]. As a consequence, increased human–animal contact and reconfigured accident occurrence patterns in urban and peri-urban areas are observed.

In Maranhão, the occurrence of accidents involving venomous animals must be analyzed in the context of high socioenvironmental vulnerability. The state presents profound structural inequalities and intense territorial transformations. In 2022, poverty and social vulnerability remained high in Maranhão, with approximately 48% of the population living below the poverty line, a proportion higher than that observed in the Northeast (41%) and in Brazil (24%), reflecting precarious housing conditions and limitations in access to basic rights [28].

Additionally, Maranhão stands out for hosting the country’s largest port complex, integrated into the logistics corridor connecting railways and highways to MATOPIBA. However, this economic dynamic coexists with high levels of territorial conflict [28]. Despite the prosperity associated with agribusiness, municipalities in MATOPIBA exhibit higher poverty rates and greater inequality than the state average, as well as a highly concentrated economy. The region encompasses 337 municipalities, with 5.9 million inhabitants and an estimated GDP of BRL 53 billion. However, more than half of this GDP is concentrated in only seven microregions, highlighting marked territorial inequality [20]. Furthermore, agricultural expansion has been accompanied by low job creation, weak local economic integration, and socioenvironmental impacts such as deforestation and territorial conflicts on Indigenous lands [20,28]. 

The interpretation of trends in this study should consider limitations inherent to health information systems. The stability observed in some Health Regions may reflect underreporting, heterogeneity in epidemiological surveillance and access to health services, as well as variation due to the small number of events in areas with smaller populations. In addition, mild accidents, such as bee stings, often do not prompt individuals to seek medical care, contributing to underreporting [24]. Part of the increase observed in certain regions may also be due to improvements in surveillance and reporting processes over time, rather than reflecting a real increase in incidence.

In this context, the One Health approach [29] is fundamental to understanding and addressing accidents caused by venomous animals, as the interactions among environmental, social, and biological factors are central to the dynamics of these conditions. Processes such as disorderly urbanization, deforestation, agricultural expansion, inadequate sanitation, and climate change act in an integrated manner [1], increasing the risk of human exposure, especially among socially vulnerable populations. Moreover, regional characteristics, such as a higher predominance of rural areas, the presence of forest remnants, the recent expansion of the agricultural frontier, and unplanned urban growth, contribute to differentiated occurrence patterns across Health Regions.

The findings of this study allow the identification of priority regions for the planning and implementation of public health actions, in alignment with the National Program for the Control of Accidents by Venomous Animals (*Programa Nacional de Controle de Acidentes por Animais Peçonhentos*, PNCAAP) [30]. These actions should incorporate the social and environmental determinants of health, considering not only incidence rates but also socioenvironmental vulnerability, access to health services, and local response capacity. Strategies such as active surveillance in critical areas, strengthening the distribution network for antivenom sera, regionally tailored training for health professionals, integrating epidemiological and environmental surveillance, and investing in basic sanitation, solid waste management, and health education are essential to reduce the impact of these conditions.

Finally, although this study is restricted to Maranhão, its results are consistent with realities observed in other Brazilian regions [5] and in tropical countries [2], where accidents caused by venomous animals constitute socially determined conditions. By highlighting temporal trends, regional inequalities, and the influence of socioenvironmental determinants, this study contributes to advancing knowledge in the field of eco-epidemiology of envenomation caused by venomous animals. It supports improving epidemiological surveillance, formulating more equitable and effective public policies within the Brazilian Unified Health System, and guiding future integrated investigations.

## Data Availability

The database used in this study is available at: https://doi.org/10.48331/SCIELODATA.UDC5BO
