# Supplementary material for: Epidemiological profile of accidents involving venomous animals: a descriptive and time series study, Maranhão, 2014–2024
Source: Epidemiol Serv Saude. 2026 Aug 3;35:e20251224. doi: 10.1590/S2237-96222026v35e20251224.en (PMC13686402; doi:10.1590/S2237-96222026v35e20251224.en)
Supplement: Translation

# Translation: Perfil epidemiológico dos acidentes com animais peçonhentos: estudo descritivo e de séries temporais, Maranhão, 2014-2024

**DOI:** 10.1590/S2237-96222026v35e20251224.pt

**Authors:** Laryssa Stefany de Azevedo Santos, Helena de Paula Gonçalves Lima, Ismália Cassandra Costa Maia Dias, Marcelo Soares dos Santos, Marcelino Santos, Leonardo Hunaldo dos Santos

**Affiliations:** 1 Universidade Federal do Maranhão Centro de Ciências de Imperatriz Imperatriz MA Brasil Universidade Federal do Maranhão, Centro de Ciências de Imperatriz, Imperatriz, MA, Brasil; 2 Universidade Federal do Maranhão Centro de Ciências Sociais Imperatriz MA Brasil Universidade Federal do Maranhão, Centro de Ciências Sociais, da Saúde e Tecnologia, Imperatriz, MA, Brasil

**Keywords:** Vigilância da Saúde Ambiental, Saúde Única, Meio Ambiente e Saúde Pública, Animais Venenosos, Epidemiologia

## Abstract

**Objetivo:**

Analisar o perfil epidemiológico e a tendência temporal dos acidentes com animais peçonhentos no Maranhão, no período de 2014 a 2024.

**Métodos:**

Estudo descritivo e ecológico com análise de séries temporais agregada por Regiões de Saúde, utilizando dados sobre acidentes com animais peçonhentos obtidos do Sistema de Informação de Agravos de Notificação (SINAN). O perfil epidemiológico foi analisado com frequências absolutas e relativas, com cálculo das taxas de incidência por grupo de animal peçonhento. A tendência temporal foi conduzida por meio do modelo de regressão linear de Prais-Winsten, estimando a variação percentual anual (VPA) com seus respectivos intervalos de confiança de 95% (IC95%).

**Resultados:**

Registraram-se 44.620 casos de acidentes com animais peçonhentos no Maranhão. O ofidismo foi o mais frequente (46,4%), com maior ocorrência em indivíduos do sexo masculino (76,3%), entre 20 e 39 anos (34,2%), autodeclarados pardos (73,0%) e com ensino fundamental incompleto (32,4%). No estado, a tendência temporal foi estável para o ofidismo (VPA 2,50; IC95% -0,10; 5,16) e para o erucismo (VPA -0,71; IC95% -3,67; 2,35), enquanto escorpionismo (VPA 7,94; IC95% 4,75; 11,23), araneísmo (VPA 7,87; IC95% 6,37; 9,40) e acidentes apílicos (VPA 4,47; IC95% 1,13; 7,92) registraram aumento.

**Conclusão:**

Os acidentes com animais peçonhentos afetaram predominantemente homens, indivíduos jovens, pardos e com baixa escolaridade. A tendência temporal indicou um cenário de preocupação crescente, sobretudo para o escorpionismo. De modo geral os achados reforçam a necessidade de vigilância contínua e de políticas públicas de prevenção regionalizadas, integrando ações epidemiológicas, ambientais e de educação em saúde.

**Aspectos éticos**

Esta pesquisa utilizou bancos de dados de domínio público e anonimizados.

## Introdução

Os acidentes causados por animais peçonhentos configuram um importante problema de saúde pública no Brasil, com implicações diretas sobre a morbimortalidade, especialmente em populações em vulnerabilidade socioeconômica. Sua ocorrência está associada a diversos fatores de risco, como o avanço de atividades humanas sobre áreas naturais e a expansão urbana e agrícola desordenada, que somam à carência de políticas públicas de prevenção e controle efetivas [1,2].

No contexto da saúde global, esses acidentes passaram a integrar, em 2007, a lista de Doenças Tropicais Negligenciadas da Organização Mundial da Saúde. Embora tenham sido retirados dessa classificação em 2013, o ofidismo voltou a ser incluído em 2017, dada sua elevada carga de morbimortalidade e a vulnerabilidade das populações afetadas [2,3].

No Brasil, os acidentes causados por animais peçonhentos são agravos de notificação compulsória, conforme diretrizes do Sistema Nacional de Vigilância em Saúde e da Política Nacional de Vigilância em Saúde [1,4]. A vigilância desses eventos é estratégica para reduzir a morbimortalidade. Em 2015, o país estabeleceu a meta de reduzir em 50% os óbitos associados a esses agravos, por meio do fortalecimento da assistência, da adequada distribuição de soros antivenenos e da organização da resposta dos serviços de saúde [1].

Entre 2007 e 2021, foram registrados aproximadamente 2.693.706 acidentes causados por animais peçonhentos no Brasil, com incidência de 88,9 casos por 100 mil habitantes. A região Nordeste concentrou parcela expressiva dessa carga, com incidência de 105,5 casos por 100 mil habitantes. No Maranhão, no mesmo período, foram notificados 44.306 casos, com incidência média de 41,13 casos por 100 mil habitantes. Fica evidente a relevância do agravo no cenário estadual [5].

O Maranhão apresenta características ecológicas e territoriais que favorecem a ocorrência desses acidentes. O estado abrange áreas de transição entre os biomas Amazônia, Cerrado e Caatinga, com forte sazonalidade climática e diversidade de usos do solo [6,7]. Nesse contexto, observa-se a presença de diversos gêneros de animais peçonhentos de importância médica, como as jararacas (Bothrops), cascavéis (Crotalus), surucucus (Lachesis), corais-verdadeiras (Micrurus), escorpiões (Tityus), aranha-armadeira (Phoneutria), viúva-negra ou marrom (Latrodectus) e aranha-marrom (Loxosceles) [1].

Muitas dessas espécies apresentam comportamento sinantrópico, isto é, adaptam-se a ambientes urbanos e periurbanos, que oferecem abrigo e alimento, o que favorece sua proliferação [1,8]. Esse risco é potencializado pelo acelerado processo de urbanização no estado. Entre 1960 e 2010, a população urbana do Maranhão cresceu cerca de 924%, acompanhada da expansão do número de municípios [9]. Associados ao desmatamento e à fragmentação de habitats, esses processos intensificam o contato humano-animal e ampliam o risco de acidentes [1].

Diante da magnitude e da heterogeneidade territorial do agravo, abordagens baseadas nas Regiões de Saúde são fundamentais para identificar desigualdades intraestaduais e subsidiar ações descentralizadas de vigilância, prevenção e atenção à saúde [10]. Nesse sentido, a análise de séries temporais constitui ferramenta essencial para identificar tendências e padrões sazonais, especialmente em agravos influenciados por determinantes ecológicos e climáticos [11].

Assim, o presente estudo teve como objetivo analisar o perfil epidemiológico e a tendência temporal dos acidentes com animais peçonhentos no estado do Maranhão, no período de 2014 a 2024.

## Métodos

### Delineamento 

Trata-se de um estudo descritivo e ecológico, com análise de séries temporais agregadas por Regiões de Saúde do Maranhão, considerando o período de 2014 a 2024.

### Fonte de dados e tratamento da informação 

Os microdados foram extraídos em outubro de 2025, por meio do sistema Tabwin, a partir dos arquivos do Sistema de Informação de Agravos de Notificação (SINAN). Os arquivos, inicialmente no formato .dbc, foram convertidos e processados no software RStudio, utilizando-se o dicionário de variáveis e a ficha de acidentes com animais peçonhentos para decodificação das observações. Os dados utilizados encontram-se disponíveis no repositório SciELO Data [13].

Não foram realizadas análises de duplicidades dos registros, uma vez que os dados disponibilizados pelo SINAN são anonimizados e agregados, não contendo uma categorização específica que permita a verificação de registros duplicados. As estimativas populacionais foram obtidas junto ao Instituto Brasileiro de Geografia e Estatística, por meio do Sistema IBGE de Recuperação Automática. Utilizou-se a população residente do Maranhão para finalidade estatística [12], com compatibilização das populações ao respectivo ano de ocorrência dos acidentes, o que garantiu a coerência temporal no cálculo das taxas.

### Contexto

O Maranhão está localizado na macrorregião Nordeste do Brasil, abrangendo área territorial de 329.651,5 km^2^ e população estimada de 6.776.699 habitantes. Administrativamente, o estado é dividido em 217 municípios [12] e regionalizado em 19 Regiões de Saúde. Estas desempenham papel estratégico na gestão do Sistema Único de Saúde (SUS), organizando, planejando e operando ações e serviços de saúde em nível regional (Figura 1) [10].

**Figura 1:** Divisão territorial do Maranhão, evidenciando a distribuição dos municípios e das Regiões de Saúde

A organização territorial das Regiões de Saúde considerada neste estudo seguiu a regionalização vigente do Maranhão, consolidada a partir das diretrizes da Resolução Comissão Intergestores Tripartite (CIT) nº 37/2018 e formalizada pela Resolução Comissão Intergestores Bipartite (CIB) nº 64/2018, que estrutura o estado nas 19 Regiões de Saúde [10]. Considerando que os registros do SINAN são produzidos em nível municipal, a agregação por Regiões de Saúde foi adotada exclusivamente para fins analíticos, não comprometendo a comparabilidade da série histórica, ainda que reorganizações administrativas tenham ocorrido no período do estudo.

### População

Foram incluídos todos os registros de acidentes com animais peçonhentos do SINAN, ocorridos entre 2014 e 2024, cujo município de ocorrência estivesse localizado no Maranhão. Os municípios foram utilizados exclusivamente para identificar e posteriormente agregar às suas respectivas Regiões de Saúde, que constituíram a unidade de análise do estudo. Foram excluídos os registros referentes à categoria “outros animais peçonhentos”, que inclui acidentes provocados por anfíbios, centopeias, cães, caprinos, gatos, equinos e outros. Essa exclusão foi adotada em razão da heterogeneidade da categoria e da limitação para análises específicas por grupo de animal.

### Período

A inclusão do ano de 2014 permitiu construir uma linha de base anterior à implementação dos Objetivos de Desenvolvimento Sustentável, iniciados em 2015. Foi possível avaliar tendências em um período ampliado. O intervalo até 2024 foi mantido para contemplar a série histórica mais recente disponível. Entretanto, reconhece-se que os anos de 2020 e 2021, correspondentes ao período da pandemia de covid-19, podem ter influenciado o padrão de notificações.

### Variáveis

Foram consideradas variáveis essenciais para a análise epidemiológica o agente animal causador do agravo, ano, município de ocorrência do acidente, sexo, faixa etária, raça/cor da pele e escolaridade. Tais variáveis apresentaram elevados percentuais de preenchimento ao longo da série histórica. A qualidade dos dados foi avaliada com base na completude das variáveis e na manutenção da definição conceitual e da estrutura de preenchimento ao longo do período analisado.

As estratificações incluídas na análise foram:

a) Ano de ocorrência do acidente (2014 a 2024);
b) Estado de ocorrência: Maranhão;
c) Regiões de Saúde (Açailândia; Bacabal; Balsas; Barra do Corda; Caxias; Chapadinha; Codó; Imperatriz; Itapecuru Mirim; Pedreiras; Pinheiro; Presidente Dutra; Rosário; Santa Inês; São João dos Patos; São Luís; Timon; Viana; Zé Doca);
d) Sexo (masculino, feminino, ignorado/em branco);
e) Faixa etária (<1, 1-4, 5-9, 10-14, 15-19, 20-39, 40-59, 60-64, 65-69, 70-79, ≥80, ignorado/em branco);
f) Raça/cor da pele (branca, preta, parda, amarela, indígena, ignorado/em branco);
g) Escolaridade (não se aplica, analfabeto, fundamental incompleto, fundamental completo, médio incompleto, médio completo, superior incompleto, superior completo, ignorado/em branco);
h) Tipo de acidente (serpente, escorpião, aranha, abelha, lagarta).

### Métodos estatísticos

A análise descritiva foi realizada calculando-se as frequências absolutas e relativas das variáveis selecionadas. As taxas de incidência anual por 100 mil habitantes foram calculadas para cada tipo de acidente e Regiões de Saúde, conforme a fórmula:


$$TI=\left(\frac{O_{URS}}{P_{URS}}\right)\;\times \;100.000$$

Onde:

TI – Taxa de incidência por 100 mil habitantes;

$O_{URS}$ – Ocorrências de acidentes com animais peçonhentos por Regiões de Saúde e ano;

$P_{URS}$ – População residente exposta por Regiões de Saúde e ano.

A análise de tendência temporal das taxas foi conduzida utilizando-se o modelo de regressão linear de Prais-Winsten, com correção da autocorrelação serial de primeira ordem (AR(1)) a partir das taxas de incidência previamente transformadas em logaritmo decimal, para cada tipo de acidente, considerando como unidades ecológicas as 19 Regiões de Saúde. 

A partir dos coeficientes obtidos na regressão, foi estimada a variação percentual anual (VPA) e seus respectivos intervalos de confiança de 95% (IC95%), segundo as fórmulas abaixo [8]:


$$VPA=\left[-1+10^{b1}\right]\;\times \;100\%$$

$${IC}_{mínimo}95\%=\left[-1+\;10^{IC\;mín.\;do\;b1}\right]\;\times \;100\%$$

$${IC}_{má\times imo}95\%=\left[-1+\;10^{má\times .\;do\;b1}\right]\;\times \;100\%$$

A tendência foi interpretada conforme os seguintes critérios [8]:

- Crescente: variação positiva, cujo IC95% não incluiu o valor nulo;
- Decrescente: variação negativa, cujo IC95% não incluiu o valor nulo;
- Estável: variação cujo IC95% incluiu o valor nulo.

Todas as análises foram conduzidas no software RStudio, versão 4.5.0, com uso do pacote prais [14].

## Resultados

No Maranhão, entre 2014 e 2024, foram notificados 44.620 casos de acidentes com animais peçonhentos. O ofidismo foi a forma mais frequente (46,4%), seguido pelo escorpionismo (40,7%), araneísmo (6,0%), acidentes apílicos (5,0%) e erucismo (1,9%). No geral, os acidentes com animais peçonhentos afetaram principalmente homens (65,0%), indivíduos com idade entre 20 e 39 anos (34,5%), autodeclarados pardos (76,7%) e com baixa escolaridade (28,8%).

O ofidismo apresentou a maior incidência (33,4/100 mil habitantes) no período analisado (Figura 2). O perfil predominante foi de homens (76,3%), adultos na faixa etária de 20 a 39 anos (34,2%), pardos (73,0%) e com baixa escolaridade (32,4%) (Tabela 1). Em relação à tendência temporal, observou-se crescimento em algumas Regiões de Saúde, destacando-se Balsas (VPA 4,04; IC95% 2,04; 6,07), Pinheiro (VPA 7,65; IC95% 4,93; 10,43), Rosário (VPA 5,38; IC95% 3,40; 7,40) e Viana (VPA 4,14; IC95% 1,68; 6,66). Entretanto, para o conjunto do estado, a tendência foi estável (VPA 2,50; IC95% -0,10; 5,^16^) (Tabela 2, Figura 2).

**Tabela 2:** Variação percentual anual (VPA) e intervalo de confiança (IC95%) da taxa de incidência (a cada 100 mil habitantes) por tipo de acidente e Regiões de Saúde. Maranhão, 2014-2024 (n=44.620)

Regiões de Saúde	Ofidismo VPA (IC95%)	Escorpionismo VPA (IC95%)	Araneísmo VPA (IC95%)	Acidente apílico VPA (IC95%)	Erucismo VPA (IC95%)
Açailândia	0,70 (-0,24; 1,64)	10,23 (7,38; 13,17)	7,24 (4,94; 9,60)	8,85 (-0,22; 18,74)	2,05 (-6,93; 11,89)
Bacabal	0,98 (-2,38; 4,45)	12,25 (5,89; 18,98)	10,80 (8,57; 13,09)	5,23 (-2,64; 13,73)	1,96 (-5,22; 9,69)
Balsas	4,04 (2,04; 6,07)	11,90 (5,06; 19,18)	8,63 (3,02; 14,54)	4,11 (-1,00; 9,49)	-5,57 (-12,44; 1,84)
Barra do Corda	3,85 (-1,71; 9,72)	6,83 (1,23; 12,74)	6,58 (0,31; 13,24)	11,84 (2,96; 21,48)	-0,28 (-7,11; 7,06)
Caxias	0,81 (-4,20; 6,09)	4,03 (1,29; 6,83)	5,02 (-0,46; 10,81)	9,71 (4,17; 15,54)	4,42 (0,98; 7,97)
Chapadinha	2,29 (-0,26; 4,89)	3,37 (0,27; 6,57)	14,45 (8,56; 20,67)	-1,28 (-3,57; 1,07)	11,26 (7,75; 14,88)
Codó	1,07 (-5,28; 7,84)	6,41 (3,24; 9,68)	8,93 (6,69; 11,22)	15,51 (10,02; 21,28)	1,62 (-4,94; 8,62)
Imperatriz	0,42 (-1,68; 2,55)	7,55 (5,67; 9,47)	11,03 (6,56; 15,70)	11,27 (6,00; 16,80)	-3,34 (-7,55; 1,06)
Itapecuru Mirim	1,70 (-0,19; 3,63)	1,67 (-0,46; 3,84)	4,76 (-0,52; 10,32)	10,75 (4,43; 17,44)	7,70 (-2,42; 18,87)
Pedreiras	-0,53 (-3,23; 2,24)	12,68 (7,03; 18,63)	6,23 (0,12; 12,71)	0,03 (-7,28; 7,92)	-2,90 (-5,37; -0,36)
Pinheiro	7,65 (4,93; 10,43)	9,38 (1,10; 18,34)	11,24 (6,48; 16,21)	2,54 (-1,64; 6,90)	-2,91 (-8,88; 3,45)
Presidente Dutra	1,00 (-3,72; 5,95)	3,76 (1,14; 6,44)	7,68 (5,86; 9,53)	6,51 (-1,74; 15,45)	-0,88 (-12,25; 11,96)
Rosário	5,38 (3,40; 7,40)	3,81 (2,19; 5,45)	4,08 (1,20; 7,05)	21,99 (14,85; 29,57)	1,53 (1,53; 1,53)
Santa Inês	1,90 (-1,49; 5,41)	12,47 (8,36; 16,74)	12,18 (6,15; 18,54)	9,26 (-6,14; 27,18)	-8,96 (-23,80; 8,78)
São João dos Patos	-0,82 (-4,04; 2,50)	6,56 (2,77; 10,49)	9,78 (3,57; 16,37)	-14,79 (-18,15; -11,30)	5,15 (-0,06; 10,63)
São Luís	3,64 (-0,21; 7,63)	6,76 (2,33; 11,39)	4,87 (2,74; 7,04)	0,15 (-5,66; 6,33)	2,83 (-4,96; 11,25)
Timon	3,73 (-1,64; 9,39)	11,68 (2,63; 21,52)	7,55 (3,33; 11,95)	-9,24 (-20,86; 4,08)	1,29 (1,29; 1,29)
Viana	4,14 (1,68; 6,66)	12,45 (6,23; 19,04)	5,14 (3,90; 6,39)	0,37 (0,37; 0,37)	4,94 (-14,39; 28,63)
Zé Doca	0,63 (-2,12; 3,46)	10,35 (3,51; 17,64)	3,04 (-2,28; 8,64)	-6,29 (-8,19; -4,35)	-24,58 (-24,58; -24,58)
Maranhão	2,50 (-0,10; 5,16)	7,94 (4,75; 11,23)	7,87 (6,37; 9,40)	4,47 (1,13; 7,92)	-0,71 (-3,67; 2,35)

**Tabela 1:** Frequência absoluta (n) e percentual (%) dos acidentes com animais peçonhentos segundo variáveis sociodemográficas. Maranhão, 2014–2024 (n=44.620)

Variável	Ofidismo n (%)	Escorpionismo n (%)	Araneísmo n (%)	Acidente apílico n (%)	Erucismo n (%)
Sexo					
Masculino	15.822 (76,3)	9.875 (54,4)	1.348 (50,1)	1.462 (65,9)	486 (58,0)
Feminino	4.904 (23,7)	8.272 (45,6)	1.343 (49,9)	756 (34,1)	352 (42,0)
**Faixa etária ** (anos)					
<1	370 (1,8)	464 (2,6)	89 (3,3)	92 (4,1)	49 (5,8)
1-4	363 (1,8)	533 (2,9)	61 (2,3)	167 (7,5)	81 (9,7)
5-9	1.158 (5,6)	881 (4,9)	95 (3,5)	209 (9,4)	119 (14,2)
10-14	1.725 (8,3)	1.087 (6,0)	117 (4,3)	153 (6,9)	101 (12,1)
15-19	2.186 (10,5)	1.463 (8,1)	175 (6,5)	180 (8,1)	45 (5,4)
20-39	7.098 (34,2)	6.241 (34,4)	1.093 (40,6)	729 (32,9)	215 (25,7)
40-59	5.458 (26,3)	4.997 (27,5)	744 (27,6)	486 (21,9)	155 (18,5)
60-64	879 (4,2)	891 (4,9)	131 (4,9)	70 (3,2)	24 (2,9)
65-69	632 (3,0)	684 (3,8)	72 (2,7)	44 (2,0)	16 (1,9)
70-79	685 (3,3)	695 (3,8)	80 (3,0)	51 (2,3)	29 (3,5)
≥80	172 (1,0)	211 (1,1)	34 (1,3)	37 (1,7)	4 (0,5)
**Raça/cor da pele**					
Parda	15.124 (73,0)	14.503 (79,9)	2.101 (78,1)	1.833 (82,6)	679 (81,0)
Indígena	1.834 (8,8)	132 (0,7)	22 (0,8)	13 (0,6)	4 (0,5)
Preta	1.716 (8,3)	1.328 (7,3)	166 (6,2)	101 (4,6)	61 (7,3)
Branca	1.336 (6,4)	1.475 (8,1)	287 (10,7)	183 (8,3)	67 (8,0)
Amarela	199 (1,0)	215 (1,2)	12 (0,4)	10 (0,5)	5 (0,6)
Ignorada/em branco	517 (2,5)	494 (2,7)	103 (3,8)	78 (3,5)	22 (2,6)
Escolaridade					
Não se aplica	1.117 (5,4)	1.331 (7,3)	188 (7,0)	355 (16,0)	167 (19,9)
Analfabeto	1.575 (7,6)	1.013 (5,6)	91 (3,4)	78 (3,5)	25 (3,0)
Fundamental incompleto	6.716 (32,4)	4.790 (26,4)	522 (19,4)	568 (25,6)	252 (30,1)
Fundamental completo	2.637 (12,7)	2.019 (11,1)	249 (9,2)	222 (10,0)	74 (8,8)
Médio incompleto	1.137 (5,5)	1.207 (6,7)	177 (6,6)	111 (5,0)	33 (3,9)
Médio completo	1.445 (7,0)	2.591 (14,3)	475 (17,7)	347 (15,6)	91 (10,9)
Superior incompleto	69 (0,3)	128 (0,7)	50 (1,8)	20 (1,0)	9 (1,1)
Superior completo	137 (0,7)	313 (1,7)	104 (3,9)	52 (2,3)	16 (1,9)
Ignorado/em branco	5.893 (28,4)	4.755 (26,2)	835 (31,0)	465 (21,0)	171 (20,4)

**Figura 2:** Incidência dos acidentes com animais peçonhentos conforme tipo de agravo: (A) ofidismo; (B) escorpionismo; (C) araneísmo; (D) acidente apílico; (E) erucismo; (F) ofidismo; (G) escorpionismo; (H) araneísmo; (I) acidente apílico; (J) erucismo, segundo Regiões de Saúde. Maranhão, 2014-2024

O escorpionismo foi o segundo agravo mais frequente, com incidência (29,6/100 mil habitantes) próxima à do ofidismo (Figura 2). O perfil sociodemográfico foi semelhante ao observado para o ofidismo, com discreta predominância do sexo masculino (54,4%), ocorrência em indivíduos adultos, entre 20 e 39 anos (34,4%), pardos (79,9%) com baixa escolaridade (26,4%) (Tabela 1). A análise temporal indicou tendência crescente para o Maranhão (VPA 7,94; IC95% 4,75; 11,^23^), com crescimento observado na maioria das Regiões de Saúde e estabilidade somente em Itapecuru Mirim (VPA 1,67; IC95% -0,46; 3,38) (Tabela 2, Figura 2).

O araneísmo apresentou menor incidência (3,5/100 mil habitantes) (Figura 2) e frequência, com distribuição relativamente equilibrada entre o sexo masculino (50,1%) e feminino (49,9%), além de maior ocorrência em indivíduos de 20 a 39 anos (40,6%), pardos (78,1%) e com baixa escolaridade (19,4%) (Tabela 2), porém com tendência temporal crescente em grande parte das Regiões de Saúde, resultando em crescimento para o conjunto do estado (VPA 7,87; IC95% 6,37; 9,40) e estabilidade em Caxias (VPA 5,05; IC95% -0,46; 10,81), Itapecurum Mirim (VPA 4,76; IC95% -0,52; 10,32) e Zé Doca (VPA 3,04; IC95% -2,28; 8,64) (Tabela 2, Figura 2). 

Os acidentes apílicos apresentaram baixa incidência (3,7/100 mil habitantes) (Figura 2) e predominância do sexo masculino (65,9%), afetando principalmente adultos de 20 a 39 anos (32,9%), pardos (82,6%) com baixa escolaridade (25,6%) (Tabela 1). A tendência foi crescente para o Maranhão (VPA 4,47; IC95% 1,13; 7,92), com aumento em diversas Regiões de Saúde, especialmente em Barra do Corda (VPA 11,84; IC95% 2,96; 21,48), Caxias (VPA 9,71; IC95% 4,17; 15,54), Codó (VPA 15,51; IC95% 10,02; 21,^28^), Imperatriz (VPA 11,27; IC95% 6,00; 16,80), Itapecurum Mirim (VPA 10,75; IC95% 4,43; 17,44), Rosário (VPA 21,99; IC95% 14,85; 29,57) e Viana (VPA 0,37; IC95% 0,37; 0,37), e tendência decrescente em São João dos Patos (VPA -14,79; IC95% -18,15; -11,30) e Zé doca (VPA -6,29; IC95% -8,19; -4,35) (Tabela 2, Figura 2).

O erucismo foi o agravo menos frequente no período analisado, com baixa incidência (1,4/100 mil habitantes) (Figura 2) e discreta predominância do sexo masculino (58,0%), acometendo indivíduos de 20 a 39 anos (25,7%), pardos (81,0%) de baixa escolaridade (30,1%), apresentando distribuição mais restrita quando comparado aos demais acidentes (Tabela 1), com tendência de crescimento em Caxias (VPA 4,42; IC95% 0,98; 7,97), Chapadinha (VPA 11,26; IC95% 7,75; 14,88), Rosário (VPA 1,53; IC95% 1,53; 1,53) e Timon (VPA 1,29; IC95% 1,29; 1,^29^) e decréscimo em Pedreiras (VPA -2,90; IC95% -5,37; -0,36) e Zé Doca (VPA -24,58; IC95% -24,58; -24,58) com estabilidade para o Maranhão (VPA -0,71; IC95% -3,67; 2,35) (Tabela 2, Figura 2).

## Discussão

Os acidentes provocados por animais peçonhentos no Maranhão apresentaram um padrão epidemiológico marcado pela coexistência de diferentes agravos. Observou-se predomínio do ofidismo (46%) com participação expressiva do escorpionismo (41%). Esse perfil reflete a diversidade ecológica, climática e territorial do estado, além da heterogeneidade dos processos de ocupação humana. A distribuição desigual dos agravos entre as Regiões de Saúde evidencia a influência de fatores ambientais, socioeconômicos e de uso do solo na dinâmica desses acidentes, reforçando a necessidade de análises regionalizadas e de intervenções territorialmente orientadas.

Do ponto de vista sociodemográfico, observou-se maior frequência de acidentados do sexo masculino e em idade produtiva. Esse padrão é amplamente descrito na literatura nacional e internacional [15,16], especialmente no caso do ofidismo. Destaca-se a relação direta com o trabalho rural exercido majoritariamente por homens (76%), predominantemente residentes na zona rural (76%), uma vez que as atividades agrícolas e o manejo manual do solo aumentam o risco de exposição às serpentes [1,17].

A maior proporção de acidentados autodeclarados pardos (76%) e com baixa escolaridade (28%) não se explica somente pela composição demográfica do estado, o que indica influência de desigualdades sociais e institucionais historicamente estruturadas. A vulnerabilidade diferencial resultante do acúmulo e da interação entre determinantes socioeconômicos passa a modular o risco de adoecimento e seus desfechos. Populações em situação de vulnerabilidade tendem a residir em áreas com moradias precárias, deficiência de saneamento básico e menor acesso aos serviços de saúde [18]. Essas condições favorecem a presença de animais peçonhentos nos ambientes domiciliar e peridomiciliar e ampliam o risco de contato humano, especialmente em contextos nos quais o acesso à informação e às medidas preventivas é limitado.

Embora cada agravo apresente dinâmica própria, todos são influenciados por determinantes socioambientais comuns. O ofidismo ocorre predominantemente em áreas rurais (76%) e está frequentemente associado a atividade de trabalho no campo [1,17]. O risco é ampliado em contextos de contato com palhadas de feijão, milho ou cana-de-açúcar, atingindo especialmente agricultores, seringueiros, pescadores, castanheiros, comunidades tradicionais e povos originários, que frequentemente residem distantes dos centros urbanos e dos serviços de saúde. Além disso, práticas tradicionais de tratamento, como o uso de torniquetes e a aplicação de substâncias caseiras (borra de café, vinagre ou urina), são comuns em relação a esses acidentes [1], o que dificulta a busca por atendimento adequado e contribuindo para a subnotificação dos casos.

No Maranhão, as principais serpentes de importância em saúde pertencem aos gêneros jararaca (Bothrops), cascavel (Crotalus), surucucu (Lachesis) e coral-verdadeira (Micrurus), incluindo jararaca-do-norte (*Bothrops atrox*), jararaca ou surucucurana (*Bothrops moojeni*), jararaca-do-rabo-branco (*Bothrops lutzi*), cascavel ou boicininga (*Crotalus durissus cascavella*), surucucu-pico-de-jaca (*Lachesis muta*), cobra-coral (*Micrurus brasiliensis*), cobra-coral ou boicorá (*Micrurus filiformis*) e cobra-coral ou coral-verdadeira (*Micrurus lemniscatus*), entre outras [1]. Apesar dessa diversidade, a relevância epidemiológica das espécies não é homogênea, uma vez que diferenças ecológicas e comportamentais influenciam o risco de contato com a população humana. Nesse contexto, a jararaca-do-norte (B. atrox) destaca-se por sua elevada capacidade de adaptação a áreas antrópicas. Ela, frequentemente, é atraída pela presença de roedores, o que favorece sua ocorrência em ambientes domiciliares e peridomiciliares, sendo responsável por 70% dos casos de ofidismo no Brasil [1].

Embora o ofidismo tenha apresentado maior número de casos no Maranhão (46%), esse padrão não se reflete no cenário nacional. No Brasil, em 2022 (183.738 casos) e 2023 (202.714 casos), o escorpionismo foi a principal forma de acidentes provocados por animais peçonhentos [1,16,19]. Em 2023 os acidentes escorpiônicos (202.714 casos) superaram amplamente os ofídicos (32.595 casos) [17, 19], especialmente em áreas urbanas densamente povoadas, que oferecem condições favoráveis à proliferação desses animais [1]. Em contraste, a maior participação do ofidismo no contexto maranhense sugere a existência de especificidades ecoepidemiológicas locais, associadas à extensa área rural do estado e à intensificação da fronteira agrícola no sul do Maranhão, região inserida no MATOPIBA (região que abrange áreas do Maranhão, Tocantins, Piauí e Bahia) [20].

A tendência temporal crescente do escorpionismo (VPA 7,94; IC95% 4,75; 11,^23^), observada na maioria das Regiões de Saúde acompanha o padrão descrito na literatura nacional. Esse comportamento tem sido fortemente associado à urbanização acelerada e desordenada [21]. No Brasil, os acidentes escorpiônicos são majoritariamente provocados por diferentes espécies do gênero Tityus [1,16]. No Maranhão, com distribuição registrada, destacam-se principalmente *Tityus obscurus* e *Jaguajir agamenon*, as quais são amplamente distribuídas nas regiões Nordeste e Norte do país [1]. De modo geral, espécies do gênero Tityus apresentam comportamento sinantrópico, elevada plasticidade ecológica e grande capacidade de adaptação a ambientes urbanos. Essas características favorecem sua proliferação em locais com acúmulo de lixo doméstico, imóveis antigos, terrenos baldios e com saneamento básico precário [22]. Tais condições são recorrentemente descritas em centros urbanos e áreas periurbanas do Maranhão [23].

No Maranhão, estão presentes três gêneros de aranhas de importância em saúde: aranha-marrom (Loxosceles), viúva-negra ou marrom (Latrodectus) e aranha-armadeira (Phoneutria). Espécies dos gêneros Loxosceles e Latrodectus apresentam boa adaptação a ambientes domiciliares e peridomiciliares, onde são frequentemente encontradas em entulhos, roupas e calçados. Os acidentes ocorrem, em geral, por compressão do animal contra o corpo. Já as aranhas do gênero Phoneutria apresentam comportamento mais agressivo e menor adaptação ao ambiente urbano. Estão associadas a áreas com maior cobertura vegetal, especialmente folhas de palmeiras e bromélias. Nesses casos, os acidentes ocorrem ativamente, enquanto elas se deslocam para forrageio e para reprodução ou quando as vítimas manipulam objetos no ambiente natural [1].

Os acidentes apílicos apresentam tendencia crescente (VPA 4,47; IC95% 1,13; 7,92) e o erucismo estabilidade temporal (VPA -0,71; IC95% -3,67; 2,35), os quais corresponderam às menores incidências observadas. As abelhas africanizadas (*Apis mellifera*) encontram-se amplamente distribuídas em áreas urbanas e rurais, e a gravidade dos acidentes está, em geral, associada à ocorrência de múltiplas ferroadas. Em 2023 os acidentes apílicos corresponderam a 8% do total de acidentes provocados por animais peçonhentos. Como a maioria dos acidentes envolve somente uma abelha, o número total desse evento pode estar sujeito à subnotificação [24].

No período de 2019-2023 o erucismo, ocasionado principalmente por lagartas do gênero Lonomia, exibiu maior incidência nas regiões Sul (5,90/100 mil habitantes) e Sudeste (4,43/100 mil habitantes) do Brasil [25], padrão associado a condições climáticas e ambientais específicas. Entretanto, processos como mudanças climáticas, desmatamento e aumento da luminosidade urbana têm favorecido a expansão geográfica dessas espécies para novas áreas urbanizadas [1,25]. Essas lagartas ocorrem tanto em áreas urbanas quanto rurais; atualmente são consideradas sinantrópicas. Os acidentes ocorrem comumente durante o contato humano com troncos de árvores próximos ao solo. A semelhança das lagartas com a casca das árvores dificulta sua identificação visual, o que favorece o contato acidental e a ocorrência dos acidentes [1].

A sazonalidade e o regime de chuvas desempenham papel relevante na ocorrência de acidentes causados por animais peçonhentos. De modo geral, observa-se maior frequência entre os meses de outubro e abril. O aumento dos acidentes ofídicos e escorpiônicos ocorre principalmente durante os períodos chuvosos, quando há maior atividade biológica desses animais [1]. Além disso, o deslocamento humano em áreas alagadas torna-se mais frequente nesses períodos, ampliando o risco de exposição.

Projeções relacionadas às mudanças climáticas e à adequação ambiental sugerem alterações na distribuição espacial e no comportamento de animais peçonhentos, principalmente de escorpiões e aranhas [26]. Modelagens climáticas indicam potenciais efeitos das mudanças ambientais sobre serpentes peçonhentas incluindo a redistribuição de áreas climaticamente adequadas. Projeções climáticas demonstram que, até 2070, diversas espécies de serpentes peçonhentas poderão perder áreas potencialmente adequadas para sua sobrevivência, enquanto ouras regiões como África e Asia poderão se tornar mais vulneráveis a ocorrência desses animais, com possíveis repercussões nos padrões de risco de acidentes [27]. Esses processos somam-se às mudanças ecológicas recentes decorrentes do desmatamento e da expansão urbana, as quais favorecem adaptações ecológicas, sobretudo em espécies sinantrópicas [1]. Como consequência, observam-se a ampliação do contato humano-animal e a reconfiguração dos padrões de ocorrência dos acidentes em áreas urbanas e periurbanas.

No Maranhão, a ocorrência de acidentes por animais peçonhentos deve ser analisada considerando um contexto de elevada vulnerabilidade socioambiental. O estado apresenta profundas desigualdades estruturais e intensas transformações territoriais. Em 2022, a pobreza e a vulnerabilidade social permaneceram elevadas no Maranhão, cerca de 48% da população vivia abaixo da linha da pobreza, valor superior às medidas do Nordeste (41%) e do Brasil (24%), o que reflete condições habitacionais precárias e limitações no acesso a direitos básicos [28].

Adicionalmente, o maranhão destaca-se pela presença do maior complexo portuário do país, integrado ao corredor logístico que conecta ferrovias e rodovias ao MATOPIBA. Contudo, essa dinâmica econômica convive com elevados níveis de conflito territorial [28]. Apesar da prosperidade associado ao agronegócio, os municípios do MATOPIBA apresentam índices de pobreza e desigualdades superiores às médias estaduais, além de uma economia fortemente concentrada. A região engloba 337 municípios, com 5,9 milhões de habitantes e PIB estimado em R$ 53 bilhões. No entanto, mais da metade está concentrada em somente sete microrregiões, o que evidencia a acentuada desigualdade territorial [20]. Ademais, a expansão agropecuária tem sido acompanhada por baixa geração de empregos, integração econômica local debilitada e impactos socioambientais, como desmatamento e conflitos territoriais em terras indígenas[20,28]. 

A interpretação das tendências neste estudo deve considerar limitações inerentes aos sistemas de informação em saúde. A estabilidade observada em algumas Regiões de Saúde pode refletir subnotificação, heterogeneidades na vigilância epidemiológica e no acesso aos serviços de saúde, bem como variações decorrentes do pequeno número de eventos em áreas com menor contingente populacional. Além disso, acidentes leves, como os apílicos, frequentemente não motivam a busca por atendimento médico, contribuindo para o sub-registro [24]. Parte do aumento observado em determinadas regiões pode ainda estar associada ao aprimoramento da vigilância e dos processos de notificação ao longo do tempo, não resultando necessariamente em elevação real da incidência.

Nesse contexto, a abordagem de Uma Só Saúde [29] mostra-se fundamental para compreender e enfrentar os acidentes causados por animais peçonhentos, uma vez que a interação entre fatores ambientais, sociais e biológicos é central na dinâmica desses agravos. Processos como urbanização desordenada, desmatamento, expansão agrícola, precariedade do saneamento e mudanças climáticas atuam de forma integrada [1], ampliando o risco de exposição humana, especialmente entre populações socialmente vulneráveis. Ademais, características regionais como maior predominância de áreas rurais, presença de remanescentes florestais, expansão recente da fronteira agrícola e crescimento urbano não planejado contribuem para padrões diferenciados de ocorrência entre as Regiões de Saúde.

Os achados deste estudo permitem identificar regiões prioritárias para o planejamento e a implementação de ações de saúde pública, em alinhamento com o Programa Nacional de Controle de Acidentes por Animais Peçonhentos [30]. Essas ações devem incorporar os determinantes sociais e ambientais da saúde, considerando não apenas as taxas de incidência, mas também a vulnerabilidade socioambiental, o acesso aos serviços de saúde e a capacidade local de resposta. Estratégias como vigilância ativa em áreas críticas, fortalecimento da rede de distribuição de soros antivenenos, capacitação regionalizada de profissionais de saúde, integração entre vigilâncias epidemiológica e ambiental, bem como investimentos em saneamento básico, manejo de resíduos sólidos e educação em saúde são essenciais para reduzir o impacto desses agravos.

Por fim, embora este estudo esteja restrito ao Maranhão, seus resultados dialogam com realidades observadas em outras regiões brasileiras [5] e em países tropicais [2], onde os acidentes causados por animais peçonhentos constituem agravos socialmente determinados. Ao evidenciar tendências temporais, desigualdades regionais e a influência de determinantes socioambientais, este estudo contribui para o avanço do conhecimento no campo da ecoepidemiologia dos acidentes causados por animais peçonhentos e oferece subsídios para aprimorar a vigilância epidemiológica e formular políticas públicas mais equitativas e eficazes no âmbito do Sistema Único de Saúde, além de orientar futuras investigações integradas.

## Data Availability

O banco de dados utilizados na pesquisa está disponível em: https://doi.org/10.48331/SCIELODATA.UDC5BO
